# Supplementary material for: Mathematical basis and toolchain for hierarchical optimization of biochemical networks
Source: PLoS Comput Biol. 2024 Dec 2;20(12):e1012624. doi: 10.1371/journal.pcbi.1012624 (PMC11637339; doi:10.1371/journal.pcbi.1012624)
Supplement: Attachment — Submitted filename: Response_To_Reviewers.pdf [file pcbi.1012624.s001.pdf]

Dear Editor,

We have carefully considered the reviewers' recommendations and are pleased to submit a revised version of our manuscript for publication in Plos Computational Biology.

The revised version has the following main changes:

we have modified the text and some figures to take into account the comments of the reviewers
we have reorganized the figures. Figure 2 and 4 became Figure 3,4,5
we have added a new figure, Figure 11 to address a question of the reviewers

In the revised manuscript, all changes are marked in red.

Sincerely,

Point by point answers to the referees. The referee's comments are in *italics*. Our responses are in red, and excerpts from the modified text are in blue.

Reviewer #1:

In the manuscript "Hierarchical optimization of biochemical networks", Viswan et al. describe an approach for the hierarchical decomposition of estimation problem. Instead of solving the full optimisation problem directly, the authors propose to construct a sequence of optimisation problems based on the topology of the biochemical process.

To tackle increasingly large models of biological processes, parameter estimation methods have to be efficient and scalable. To achieve this, a variety a novel concepts have been proposed, including hierarchical optimisation strategies for observation and noise parameters. Here, the authors propose to nested hierarchical optimization to improve parameter estimation further. Unfortunately, the propose is not compare to state of the art method and a discussion of related approach is missing. In my opinion, the extended case with feedback appears similar to the dependent input approach by van Riel and Sontag (DOI: 10.1049/ip-syb:20050076). Furthermore, the paper would profit from an assessment of limitation and applicability, and a comparison further comparison with state-of-the-art methods (see comments below).

We thank the reviewer for these useful and constructive comments.

Major:

1) The manuscript should discuss related approaches, e.g. the method by Kotte & Heinemann (https://doi.org/10.1093/bioinformatics/btp004) and van Riel & Sontag (DOI: 10.1049/ip-syb:20050076) which also uses a decomposition of the overall problems. As these methods have not been widely used, it would be important to discuss why the authors believe that this will be different for their approach.

We thank the reviewer for suggesting these references and have incorporated them, with the statement:

Decomposition of optimization problems has been previously explored for hybrid Petri Net models of signaling \cite{koh2006decompositional}, using autonomous modules, though without extending the approach to networks with feedback. In cases involving sparse data, under more restrictive conditions, the decomposition of objective functions into a sum of independent terms has been proposed as a way to split optimization into simpler independent problems \cite{kotte2009divide}. Decomposition in the presence of feedback has also been addressed through the method of dependent inputs \cite{van2006parameter}.

Hierarchical optimization has been applied to the optimization of systems biology models, particularly in its simplest form, known as bilevel optimization \cite{loos2018,schalte2022efficient}.

In this paper, we present a comprehensive mathematical and algorithmic solution for the hierarchical decomposition and multilevel hierarchical optimization of general networks, including feedback, and implement it in software. Our toolset employs JSON format specifications for both optimization configuration and experimental datasets, ensuring easy interoperability and reusability, even across toolsets with different syntaxes.

2) As there are already hierarchical approaches published for ODE models of biochemical processes (e.g. reference 25 which you include), the author should in my opinion choose a much more specific title. Furthermore, the reference 25 and its extensions in several directions (see for a summary: https://mediatum.ub.tum.de/doc/1625172/a5r8m48zdiyp4qhwmhuqrylg2.PhDThesis_YannikSchaelte_20220725.pdf) should in my opinion be mentioned in the introduction.

We thank the reviewer for the suggestion and now utilize the title “Mathematical Basis and Toolchain for Hierarchical Optimization of Biochemical Networks “ We have also mentioned the suggested thesis, of which we were not aware. At the end of the Introduction we have regrouped all the present approaches, with the statement

Decomposition of optimization problems has been previously explored for hybrid Petri Net models of signaling \cite{koh2006decompositional}, using autonomous modules, though without extending the approach to networks with feedback. In cases involving sparse data, under more restrictive conditions, the decomposition of objective functions into a sum of independent terms has been proposed as a way to split optimization into simpler independent problems \cite{kotte2009divide}. Decomposition in the presence of feedback has also been addressed through the method of dependent inputs \cite{van2006parameter}.

Hierarchical optimization has been applied to the optimization of systems biology models, particularly in its simplest form, known as bilevel optimization \cite{loos2018,schalte2022efficient}

3) L. 18-21: "In particular, parameter optimization methods have been implemented in a fragmented manner [9–17]. This is in part due to the very wide diversity of experimental inputs used to constrain such models, but also due to the inherent contradictions and incompleteness of the parameter constraints." - It is unclear for me in which regarding the authors considered the implementation of optimisation methods to be fragmented. There are various comprehensive

optimisation packages with a broad support, and even in systems biology there are toolboxes such as COPASI, D2D and PyPESTO supporting dozens of optimisers which allow for constraints.

We thank the reviewer for bringing this up, so that we may clarify. In the revised text we now say:

Parameter optimization frameworks have been implemented in a diverse manner, with different specification formats for the parameters, the experimental datasets, the parameter bounds, the objective functions and the choice of optimization methods.

We also cite PyPESTO, COPASI and PETab

4) L. 87-88: "In the HOSS calculations we perform two levels of scoring. First, for each experiment for which the sub-model is tested" - At this point submodes have not ben formally introduced. I would recommend to change the order and to start with a comprehensive model formulation and a definition of submodes, before specifying the objective function.

We thank the referee for this remark. In fact, the Eqs. 2,3 apply both for submodels and models. We therefore change submodels by models because submodels are not yet introduced.

5) L. 87-98: In my opinion the use of statistical interpretable objective functions is highly beneficial. For the objective function used by the authors for an individual dataset (Eq. 2), corresponds to the case with additive normally distributed measurement noise with mean zero and standard deviation equal to the maximum of the observed output. This on its own might be questions, but the interpretation of the sum become even more problematic (Eq. 3).
It would be interesting to know why the authors did not chose an objective functions which allows for statistical meaningful uncertainty quantification.

We thank the referee for this question.

We agree with the referee that it is beneficial to have statistically interpretable objective functions. However, the main role of the objective/loss function is to assess training error. While the formulation in Eq. 1 has statistical significance, its main advantage in our context of integrating multiple experimental results lies in the rescaling the various contributions to the objective functions, which is done differently in Eqs. 2 and 3. Indeed, it is difficult to find a correct statistical model for the variance resulting from experiments performed by different laboratories and under different biological conditions.

We have explained this decision in more detail in the revised text:

We used this simple way to rescale the terms in the least-squares objective function because the highly heterogeneous nature of the experimental datasets does not lend itself to mathematically expressed statistical quantification of uncertainty. Specifically, different experiments disagree (e.g., Figure5C) and are performed under different biological contexts. While some experiments do provide error bars, there is no rigorous way to combine such estimates when the main source of difference between readings is not measurement noise, but details of the experimental system being employed. We instead chose to incorporate a semi-quantitative expert assessment of data reliability by assigning weights to each experiment used for estimating the optimization cost. Our experimental data format (FindSim format, \cite{viswan2018findsim}) includes error estimates for

future use of more elaborate objective functions in cases where datasets are more homogeneous.

6) L. 103-105: "we use multistart optimization, by launching local search procedures from randomly chosen starting points generated uniformly in logarithmic scale" - It has been shown that using log-transformed parameters for optimization (not only initial sampling) improve performance tremendously (https://doi.org/10.1093/bioinformatics/btz020 and your reference [26]). It would be interesting to see what can be gained here.

We thank the referee for this question. In the text we have clarified the difference as follows:

Similar to \cite{villaverde2019benchmarking}, which uses a logarithmic scale for both initial and evolving parameter values, we allow an option between logarithmic and linear scales for the optimizer, with logarithmic as the default.

7) Eq. 6: The readability of Eq. 6 could in my opinion be improved. I would recommend to stick to standard terminology and to defined the optimal points directly, e.g. p_{K-1}^* = \arg \min_{p_{K-1}} {...}, and to use "subject to" to refer to the conditions in the next level.

We thank the referee for this suggestion.

We believe that our notation is more concise and appropriate, given the context. Indeed, in the first of the equations (6) p^*_{K-1} is a solution (solves) the optimization problem defined in the second line. The solutions can be multiple, and, in the next lines, depend on the non-optimized parameters. Our choice is the same as the one used in the ref 22.

8) L. 154-156: "1) if a species is in the subset I, then all the reactions consuming or producing this species are in the corresponding reaction subset J, namely if i \in I then j \in J whenever S_{ij} \neq 0.$ - The statement "if i \in I then j \in J whenever S_{ij} \neq 0" implies in my opinion that all species in I are influences by all reactions in J. This is not what the first part of the sentence states and seems to be awfully restrictive. Please check

We thank the referee for this warning.

In fact, there is no issue here. The implication is i \in I \implies j \in J if S_{ij} \neq 0. This means that if a species i is in I than all the reactions that consume or produce this species are in J. However, thIs does not mean that every reaction in J will act on all the species in I. In other words the reciprocal implication is not necessarily true.

9) Conceptually, the nested decomposition results smaller subproblems. Yet, the issue is that the inference on the small module does only consider the data which can be directly mapped to the module. Consider the reaction network R1: A -> B with rate k1*A, R2: B -> C with rate k2*B, and A and B being measured. The method would infer a small module mit A and R1 only from data in A, but clearly measurements of B provide additional information, in particular if they have a higher resolution.
It would be interesting to assess how much information os lost.

We thank the referee for this interesting comment.

In nested hierarchical learning the data is used progressively starting with part of the data to solve a simpler problem, just like the referee has noticed it. For this reason we would like to avoid having too small blocks in the decomposition. In practice, this is performed by the choice of the parameter r in the r-SCC partition (if the blocks are too small, one has to increase r).

10) The formulation of the nested decomposition depends on the existence of a directionality in the graph (as shown in Figure 1). I assume that many models cannot be decomposed. Did the authors show for which fraction of models a nested decomposition is possible? The could be easily done for the Biomodels database and would -- if the fraction is high and the models are substantial is nice -- increase the impact of the contribution. Even better would be the evaluation of the PEtab benchmark collection taking also the data mapping into account.

We thank the referee for this question.

In signaling networks, directionality is present because the function of these networks is to transform an input signal into a cellular decision, mediated by changes in effector proteins. Although feedback mechanisms can blur the trails, directionality remains a key feature in signaling. The question is very interesting for CRNs in general, and warrants a more detailed response, as it is not straightforward. In fact, we are preparing a comprehensive answer to this question in a separate paper, which includes benchmarking of Biomodels and the details of the hierarchical decomposition.

However, using r-causality and agony allows the decomposition of any chemical reaction network. The figures below summarize the result of benchmarking our decomposition algorithm on the 33 models of the PEtab collection.

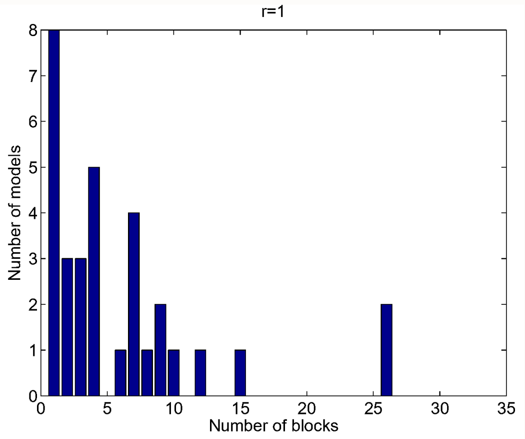

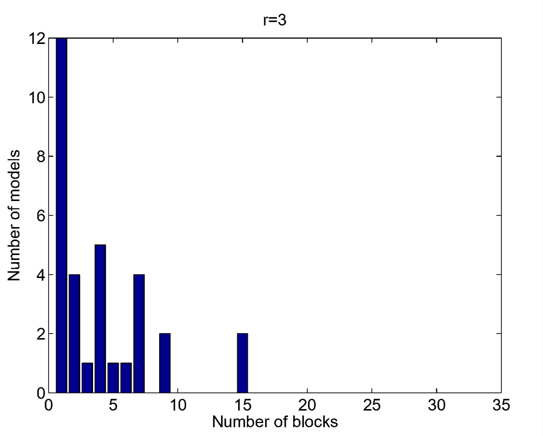

For small values of r, the algorithm decomposes the models in small blocks. 25 from 33 models in the PEtab collections are decomposed in more than one block for r=1.

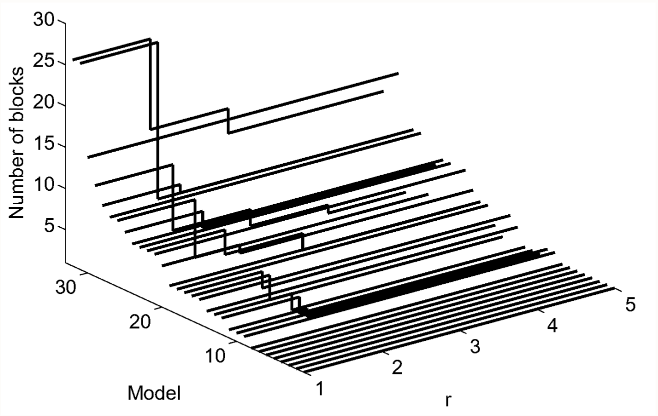

Increasing r, decreases the number and increases the size of the blocks. Many models have several blocks even for large r. The models are sorted by the number of blocks for r=1. The figure does not include the model Froehlich_CellSystems2018 that has 1396 species and 252 blocks (for r=1), 238 blocks (for r=10).

11) L. 333-334: "For the purposes of this report, we model two signalling pathways in two formalisms each (Figure 4 A-D)." - I would have preferred if already published models were used. The formulation of own models and datasets raises the questions if they are tailor-made for the proposed approach.

We agree we should have provided the provenance of the models. This is now given. We also explain that there was no human selection process involved in choosing the experimental dataset for these particular models. Like other published models, our models were generated to address specific biological questions and not to accommodate the proposed approach in any way. We added the following text

The ODE models are subsets of the model presented in (Bhalla and Iyengar 1999), DOQCS accession 3 (https://doqcs.ncbs.res.in/template.php?&y=accessiondetails&an=3). They have been modified slightly as part of the subsetting process. The HillTau models were derived from the ODE models by abstracting the reaction steps. The selected experiments were obtained programmatically from an in-house dataset by selecting for those experiments having stimulus and readout molecules that were present in the models. All models and experiment files used in the optimization are provided on GitHub and were generated to answer specific biological questions in neuroscience independent of the current study.

12) L. 348-349: "the number of experiments pertaining to each pathway is limited, and considerably below the number of parameters" - In my intuition this should be a very problematic setup for the proposed method. If a submodel is non-identifiable based on the corresponding data, parameters might be chosen which do not allow to achieve good estimates in subsequent iteration. I would appreciate if the authors could comment on this to complement the section of degeneracy.

We thank the referee for bringing this up.

In biological and medical applications, fitting very complex models with marginally sufficient data, is far from being an exception and is by no means a peculiarity of our method. We have addressed this issue in the subsection model degeneracy, considering several parametric solutions. In the hierarchical framework we can perfectly deal with this using the hossMC method. In this method, at each hierarchical level we consider optimal solutions together with suboptimal ones and these are propagated through the hierarchy. This allows us to keep track of the parametric degeneracy.

13) L. 386-387: "As a reference, we first ran the HOSS pipeline using flat (non-hierarchical) optimization on the models, employing a number of standard optimization methods in the scipy.minimize library (Figure 7 A)." - For a meaningful comparison, state of the art methods which are tailored to the problem class should ideally be used. For ODE models, these approaches are for instance implemented in D2D, PEtab.jl and pyPESTO. In particular, for gradient based optimisation, sensitivity equation based formulation should be used.

We thank the referee for this suggestion. We have already compared the standard optimization algorithms in Python. Hoss is an evolving environment, and we plan to make our software open to the implementation of new optimization functions, including those proposed by the referee. We believe this is an excellent idea and will certainly incorporate it in future versions of our software.

Minor:

L. 21-28: I see the problem mentioned here, but I do not see how this supports / ore is even connected to the previous statements in the paragraph or the contributions of the paper in general.
Indeed, the authors seem to fall in the same trap (see line 345-348).

We have clarified the point with an additional line:

Our framework systematizes how experiments are used for optimization, and provides a way to assign a numerical confidence in each, thus ensuring that optimization runs are reproducible and can evolve as better experiments become available.

L. 32-34: "The current paper focuses on standardizing the calibration and optimization stages of model development, given a large but incomplete set of experimental data." - In my opinion there is nothing like a "complete dataset". I guess the authors want to refer here to having only partial observation, but this should be clarified. Furthermore, it would be interesting to know how the authors see the work being related to the establishment of the PEtab format (https://doi.org/10.1371/journal.pcbi.1008646).

We thank the reviewer for this comment. We have modified the text as follows

given large, but sparse and partially conflicting experimental datasets

We have globally replaced “incomplete” by “sparse”
L. 60-61: "We illustrate its use on an extant database of over 100 experiment definitions" - The

term "experiment definitions" is not well define and should be clarified to avoid the impression that the dataset is much larger than for other models.

We have rephrased as “A database of over 100 experiments encoded into the FindSim format”

L. 66: "parametric optimization problems" -> "parameter optimization problems"

done

L. 101: "S is a space of constraints" - S does not seem to describe the constraints but the set of admissible parameter vectors. Furthermore, "S" is used for multipel different thing. I would suggest to chose here a different symbol to avoid confusion.

We thank the referee for this suggestion. The space of constraints defines the feasible region or domain within which solutions must lie. It is a frequent word in optimization theory . To avoid confusion with the stoichiometric matrix we change S into C everywhere this means space of constraints.

L. 111-112: "We refer to this procedure as parameter scrambling. Despite its simplicity, multistart optimization with logarithmic sampling has proven to be effective in benchmarks of biochemical pathways [26]." - The reference appears inappropriate as [26] optimises xi = log(p) (using p = exp(xi) in the model) and does not only sample the starting points differently.

We thank the reviewer for this remark. We clarified this in the text by adding

Similar to \cite{villaverde2019benchmarking}, which uses a logarithmic scale for both initial and evolving parameter values, we allow an option between logarithmic and linear scales for the optimizer, with logarithmic as the default.

L. 150-151: "Some species forming a subset BS ⊂ S are buffered, and their concentrations are kept constant." - This statement is not clear for me. Are the authors referring to conserved quantitates? If so, "buffering" might not be the right term.

We use buffering in the chemical sense. In chemistry, a buffer is a species present in very large amounts, whose concentration is practically constant, despite its participation in reactions. As a result, a buffered molecule is not conserved in the simulation.

L. 172-174: "A pair of species (i, j) ∈ E defines an edge from i to j if and only if there is a reaction that consumes or produces the species j, and its rate depends on the concentration of the species i." - Please mention here or in the sentence before that you are considering direct graphs?

Actually the word digraph, a common shorthand for directed graph was used in the line 171.

L. 378: "Black-box, non-gradient optimization methods work well for flat optimization" - I'm puzzled by this observation. In our experience in particular for problems with relative flat

objective functions, gradient-based methods are fare superior. Yet, this only holds of gradients are evaluated accurately, e.g. using forward sensitivity analysis. The dependence of results on such choices could be discussed.

We thank the referee for this remark. All gradient-based methods will have problems in ill-conditioned situations that we invoke. We do not exclude that these may be milder when gradient is more precisely calculated (this would replace a noisy value by a very small one demanding more iterations but eventually leading somewhere). We therefore added to the text

We have not tested here more precise methods to estimate the gradients, such as forward sensitivity, that may have good performance even with flat cost functions. These will be implemented in future versions of HOSS.

L. 426: "Multistart methods yield lower optimization cost: initScram method" - I recommend to avoid the term "optimization costs". I would used standard terms such as "objective function value" to avoid the confusion with the "computational cost".

We followed this recommendation. Optimization cost was globally replaced by cost function value.

Reviewer #2: This is a well written paper addressing an important problem - optimization of signaling pathway models. The introduction nicely reviews the current state of the art, and explains why the problem is critical.

We thank the referee for his kind comments and interest in our paper.

My main comments relate to definition of the hierarchies, autonomous pairs and SCC.
Specifically:
Lines 172-173: sounds as if i is causal to j, but then in lines 174-175 you define causality with an example of j causal to i. I think it would be less confusing to use the same causality in both sentences.

We thank the referee for this warning.

There is no explicit contradiction but in the first example i is causal to j, whereas in the second example j is casual to i. However the first example is used to introduce the edges. An edge is a path, so i is causal to j in the first example.

To avoid confusion, we simply permute i and j in the definition of causality

From the definition of SCC on lines 178-179, I would label A, D and E in Fig 1 as SCC. However, this definition is different than you give in Figure 1. In caption to Figure 1, it seems to me that the definition of SCC doesn't apply to b, which is only connected to c and f, but not a, d or e. So, either you need to change this sentence or remove b from being a scc. Possibly this applies to some other components.

We thank the referee for this warning.

Indeed, it is implicitly considered that all species are self causal, b is a SCC. For simplicity, self interactions are not represented in the graphs. We have added this information to the caption of Figure 1.

Also, can you label autonomous pairs in this graph?

We thank the referee for this suggestion.

The species of autonomous pairs can be indicated. This information is now given in the caption. We have also relabeled the pairs starting from 0, to make lines 180-204 more readable.

The definition of J1 (line 191) is confusing. How can it include reactions _producing_ species from I1 if I2 has no incoming connections?

We thank the referee for pointing this out.

I think that the misunderstanding comes from labeling of autonomous pairs starting with 1 instead of 0. I2 has incoming connections from I1. We hope that with the relabeling and with the example of Figure 1 fully worked out, the concept becomes clear.

Fig 2 and Fig 6: I thought I understood how levels were defined, but these figures calls that into question. How is the pink block with RGR, Ras_GDP of rank 0 since it has an input. In Fig 6, how can a block be of multiple levels? I thought level 1 meant no inputs, which means it cannot be level 2. This needs to be clarified. Also, what is the difference between rank and level? They seem the same, in which case only one of those terms should be used.

We thank the referee for pointing to these issues.

Rank and level mean the same thing. For uniformization we keep level everywhere. We noticed some inconsistencies in the Figure 4 and redraw it. The same information can be now found in the Figures 3,4 and 5. Concerning the level 0 of the RGR block this is normal. This block is contained in a large loop of the quotient graph. In such a situation there are multiple labeling solutions: each of the blocks of the loop are equivalent, but for biological reasons we choose one of them (the one that receives external signals) to have level 0. We emphasize this point in the text as follows:

In cases where block labeling has multiple solutions, such as in cycles, use biological information to define as roots the blocks that receive extracellular signals.

We have corrected the inconsistencies in the Figure 6. A block does not have multiple levels, but contributes to autonomous pairs of different levels. A species subset in an autonomous pair contains all blocks of level smaller or equal to a given one. The new figure is Figure 7.

We apologize for the inconvenience.

Minor comments:

In describing types of models in the introduction, the authors leave out stochastic implementations of biochemical reactions. I'm wondering why these are excluded.

We thank the reviewer for pointing this out. We restate the line as follows:

These models take many different formalisms, including visual representations of data, such as protein interaction networks [1], and executable models like with kinetic rate laws which can be solved with ODEs or using stochastic calculations [2], boolean models [3], and more recently, HillTau abstractions [4].

Line 243 - explaining agony: If r has to be not too small to avoid r-blocks with 1 species, why do the authors use models with r equal to one?

We thank the referee for this question.

Actually, the blocks are large enough even for r=1. The number and size of blocks are quite robust so in this case it is enough to take r small enough. We explain the situation in the text.

This is because, even for $r=1$, the blocks are large enough for these models, so we typically use small values of r.

Line 266: There appears to be a verb missing in this clause

We thank the reviewer for pointing this out. We rephrase as

and find all motifs that share the same enzyme

Line 348-349: In quantifying number of experiments and demonstrating parameters are under-constrained, does that take into account that some experiments measure multiple time points? It should, since comparing multiple time points doesn't increase number of parameters, though I still expect the parameters to be under-constrained.

We thank the reviewer for pointing out our lack of clarity here. It is indeed the case that time-series experiments and dose-response experiments usually have multiple sample points. While occasionally these do provide additional constraints on the parameters, they are more often useful as ways to tighten a single parameter because they are simply successive points on an asymptotic curve. We have rephrased the statement:

First, the number of experiments pertaining to each pathway is limited, and even though some experiments provide multiple sample points (Table 1), the constraints remain considerably below the number of parameters even for HillTau models. For instance, many time-series and dose-response curves are asymptotically converging. Hence they may have many points but only constrain a single parameter.

FIg 4: Text in this figure needs to be much bigger.

We have redrawn this figure using larger fonts. The new figures are Figures 4 and 5

Reviewer #3:

Summary:
The manuscript "Hierarchical optimization of biochemical networks" by Viswan et al. introduces HOSS, a method to break down large system models into individual pathway blocks organized in a nested hierarchy, allowing for efficient parameter optimization at each level. However, there are several key issues that limit the overall usefulness of the work. One of the key issues is that it is unclear how the hierarchical decompositions handle a signaling network with significant feedback mechanism. In my opinions, feedback mechanisms are almost always present in biological signaling pathways and play crucial roles. If HOSS can only effectively break down system without feedback, it significantly diminishes the utility of the method.

We thank the referee for pointing to this issue.

The methods section of our paper addresses the hierarchical decomposition of models, regardless of whether they include feedback. Particular effort was made to cover cases with feedback, leading to the introduction of new concepts such as r-strongly connected components. Therefore, HOSS can decompose systems even in the presence of feedback. For this purpose, we use a classical method that involves replacing time-dependent inputs with experimental data when necessary (the dependent input method)..

It is possible that we have not sufficiently explained the applicability of our approach.

We have added additional text and a Figure to clarify this point, see below.

Questions:
In line 210, authors wrote, "The autonomy of these resulting blocks is only approximate, but it allows us to benefit from hierarchical optimization." What exactly does HOSS do to approximate signaling pathways with feedback? Does HOSS ignore the feedback mechanisms, break down the signaling pathways, and optimize the parameters in each block independently, then reintroduce the feedbacks to estimate their parameters, fix those feedback parameters, and then re-optimize the parameters in each block, iterating this process?

We thank the referee for this remark.

We agree that this formulation is confusing. We replace it with :

Some blocks are no longer autonomous in the presence of feedback, because they receive input from higher level blocks. In order to compute the ODE solutions and objective functions, we use a standard approach known as {\em dependent input} in systems biology\cite{van2006parameter}: the time dependent signal coming from higher level blocks is replaced by experimental data. This method is summarized in the Figure..

What are the specific benefits of hierarchical optimization when feedback mechanisms are present? Apart from reducing Wallclock time, which is not a major concern for me since estimating hundreds of parameters typically takes a few hours at most, what other advantages does this approach provide?

We thank the referee for bringing this up.

Execution time is always a concern. First, the execution time can increase dramatically (see our other remarks below on phase transition problems). Second, it is always finite, but for many

starting points, the optimization may fail to converge, often reaching values far from the optimal solution. Finally, the same model might need to be optimized multiple times, such as during cross-validation, or several different models might need to be optimized and compared using the same dataset. Therefore, any reduction in execution time is highly beneficial.

For example, in Fig 1., if there are two feedbacks loops from F3 to B0 and A0, can HOSS still break down the signaling diagram? How would HOSS approach optimizing the parameters in this scenario?

We thank the referee for this question.

As mentioned, HOSS deals with this by using the method of dependent input. For instance, the models in Figure 2 have feedback and they are still decomposed using r-SCC and agony. For the simulation and optimization the feedback is taken into account by considering the experimental data (dependent input).

If the feedback in the diagram is critical to the system and my goal is to estimate the parameters within the feedback loops, what are the advantages of using HOSS? How does the HOSS approach differ from a flat optimization approach is this context?

We thank the referee for this question.

As mentioned, HOSS can handle feedback, and the optimization problem is decomposed into simpler subproblems even when feedback is present.

Nonlinear optimization and constraint satisfaction problems become increasingly complex as the size of the problem grows. In some cases, one can encounter phase-transition situations, where the complexity surpasses a certain threshold, rendering the problems unsolvable, even with supercomputers (see, for example, the Chapter 14 of the book *The Nature of Computation* by Moore and Mertens and the paper by Achlioptas, Naor & Peres in *Nature* 435, 759–764 (2005)). Although phase transitions were not observed in the models examined, these phenomena could happen for larger models and more constraining datasets. We do however observe a steep increase in complexity with the size of the model which also justifies the modular approach.

For these reasons, modular approaches consisting in decomposing the full problem into simpler problems, are advantageous in optimization.

Another issue concerns the reaction rate vector. Are the reactions limited to zero-order, first-order, or second-order reactions? Are the reaction rates constrained to be the product of reactants with the parameters?

We thank the referee for this question.

In brief, HOSS does not restrict the order of reactions and we use standard mass-action and Michaelis Menten formulations for the kinetic laws. We also show that HOSS works well with more abstract HillTau representations based on Hill Kinetics with modifiers. In the theoretical part of the paper, we have used no assumption on the type of kinetic law.

There is a subsection about “Signal back-propagation, reduced Michaelis-Menten mechanisms,

and irreversible reactions." The authors state in line 259, "As signaling pathways models often assume QSS, it is useful to have a tool that reduces mass action models by eliminating complexes." Michaelis-Menten mechanisms can be important. Can HOSS retain the equation format for these mechanisms? For example, if parameters like ki+, ki-, and kcati are crucial for estimation based on the data, is HOSS capable of estimating these parameters, or does it resort to flat optimization in such cases?

We thank the referee for this question.

We recognize that we did not sufficiently describe the range of parameters that are optimized. Yes, HOSS can optimize all the relevant parameters. We have added the information to the text.

Although QSS reduction or forward irreversibility can improve hierarchical decomposition by decreasing the size of some modules, these two approaches are optional in HOSS. r-causality alone, or in combination with agony, can be used to identify sufficiently small modules. Furthermore, QSS reduction is applied only during the module definition phase and not during simulation, which can implement the full mechanism.

In addition to Michaelis-Menten kinetics, other rate equations, such as Hill functions and sigmoidal functions, can be used to describe reaction rates. Can HOSS incorporate these diverse rate equation formats and estimate the parameters within these equations?

We thank the referee for this question.

HOSS can incorporate diverse kinetic laws, see our answers above.

In Figure 3F, it seems that HOSS does not fit the experimental data.

We acknowledge that this needs to be clarified. Here we show one plot out of an optimization that included multiple experiments with different profiles, as well as multiple other experiments. The presented plot is one out of a large number of experimental constraints that comprise this parameterization problem, and hence the fit to this plot may have been worsened to improve the overall optimization cost.

In the caption of the Figure we added

Note that these are just two of several experiments contributing to the optimization cost function, hence the fits may not be close in every experiment.

In Figure 6C, it appears that the Flat approach yields a cost value of about 8, while the Hierarchical approach gives a cost value of around 2. However, it's difficult to determine whether the difference of approximately 6 is significant. The authors should plot the simulation results from both the Flat and Hierarchical approaches alongside the experimental data. This would illustrate what a cost value difference of 6 looks like and clarify whether HOSS offers a real advantage over flat optimization.

We thank the Reviewer for this suggestion

We modified Figure6 (now Figure 7) as suggested by the Reviewer, plotting part of the data for illustration. The cost values were not normalized and we provide the correct normalized values by changing the scale of Figure7C. In addition, we have performed a comparison of the distributions of cost value between HOSS and Flat approaches for multi-start methods with 200 starting points, for each model (Now Figure 11). From this we can see how the cost value distributions differ.

In Figure 8D, HOSS optimization is compared to Flat. In D3_EGFR case, Flat performs the same as HOSS, if not slightly better. In D4_EGFR, Flat outperforms HOSS. In D3_b2AR and D4_b2AR, HOSS shows better results than Flat. Based on these outcomes, I don't see significant benefits in using HOSS over Flat, especially since the improvement in cost value by HOSS is less than 0.05. I'm unsure how meaningful a 0.05 improvement is in this context.

As mentioned earlier, the authors should plot the simulation results from both the Flat and HOSS approaches alongside the experimental data. I suspect that a 0.05 improvement in cost value may be indistinguishable when the simulation results are plotted.

The same issues apply to Figure 10B. How significant is an improvement in cost value of less than 0.1 when comparing Flat and HOSS? The authors should plot the simulation results from both the Flat and HOSS approaches alongside the experimental data to demonstrate the importance of a cost value improvement of less than 0.1.

We thank the reviewer for these questions. We have generated more data to address the issue in a rigorous, quantitative way. These results are illustrated in the new Figure 11. Concerning visualization of the simulation results we have done this for the outcome of one of the model optimizations, since there are over 40 experiments to compare.

We added the text

To directly compare the performance of HOSS with the flat method, we ran multi-start optimizations for all four models using the two methods. We generated 200 initial models in each case, and recorded distributions of solution time and of initial and final cost functions (Figure11). The peak of the HOSS solution time was around one-third that of the flat method (Figure11A-D) except for the D3 EGFR model, where there was only a small improvement. Likewise, the cost functions for the HOSS optimization were better than those for flat optimization, except for D3 EGFR, in which case they overlapped (Figure11E-F).

Overall, if the HOSS cannot preserve feedback loops and limits the type of rate functions, but fails to provide a substantial improvement in cost value, it is difficult to justify its advantage over a straightforward flat optimization method.

We hope we have adequately conveyed that HOSS preserves feedback loops and is compatible with various types of rate functions. The numerical experiments illustrate a gain in execution time and in precision. The theoretical justification for the superiority of modular approaches over direct methods lies in the steep (much more rapid than linear) increase in optimization complexity as problem size grows.
