# Supplementary material for: Mathematical basis and toolchain for hierarchical optimization of biochemical networks
Source: PLoS Comput Biol. 2024 Dec 2;20(12):e1012624. doi: 10.1371/journal.pcbi.1012624 (PMC11637339; doi:10.1371/journal.pcbi.1012624)
Supplement: Attachment — Submitted filename: response_to_reviewers2.pdf [file pcbi.1012624.s002.pdf]

Reviewer #3: The authors have addressed most of my questions, but two remain unclear to me.

1. The first issue is regarding Figure 3F, which has been changed to Figure 2F in the revised version. In my view, HOSS does not fit the experimental data. The authors explained that “Here we show one plot out of an optimization that included multiple experiments with different profiles, as well as multiple other experiments. The presented plot is one out of a large number of experimental constraints that comprise this parameterization problem, and hence the fit to this plot may have been worsened to improve the overall optimization cost.” However, there are only two plots showing the fittings, Figure 2E and 2F. The authors need to provide the fitting results for several other “experiments with different profiles, as well as multiple other experiments” to support their explanation. I suggest adding about four panels showing different well-fitted protein level changes in addition to aEGFR and aMAPK. The authors need to provide fitting plots demonstrating which proteins are associated with the worsening of aMAPK, rather than vaguely referring to “multiple experiments with different profiles” or “multiple other experiments”. If only two plots are shown, it suggests that HOSS cannot fit both proteins simultaneously.

We thank the reviewer for this remark. Figure 2 is intended to illustrate the workflow within the pipeline and is not meant to present results, particularly in terms of assessing training accuracy.
In fact, Figure 2 contains three, not two examples of fitting, corresponding to our 3 types of experiments: bar charts, time series and dose response. We have updated the figure to use the post-optimization plots, which fit more closely.
In response to the reviewer’s suggestion, we have included two additional experiments among which there is a third molecular readout, internalized EGFR. The updated figure has five examples.
We hope the reviewer agrees that a single model cannot be perfectly fitted to data from different laboratories, as there may be conflicts within the datasets. Accordingly, we have added the following sentences to the text on page 11 under Experimental Database:
“Due to such conflicts within the datasets, a single model cannot be perfectly fitted to data from different laboratories. As a result, some species in some experiments exhibit larger training errors, despite the overall optimization cost being low.“

2. The second issue concerns Figure 7D. This plot confirmed my concern about the meaningfulness of the cost value improvement. In my view, there are no different in the fittings between the plain and hierarchical method for Erk2-pp and Mek1-pp. The hierarchical method might be slightly better than plain in Mos-P, as it avoids the incorrect upward trend. The authors also provided a new Figure 11 showing the distribution of cost values. D3_EGFR and D4 EGFR are nearly identical between HOSS and Flat methods. In the case of D3_b2AR and D4_b2AR, the HOSS method may show an average cost value improvement of 0.1, as shown in Figure 12B. Figure 7D is the only plot comparing the HOSS and Flat methods with the data. Although there may be a 0.1 difference in cost value as shown in Figure 11, Figure 7D demonstrates that this improvement is nearly indistinguishable when the simulation results are plotted. I suggest that author select 3 additional proteins that best highlight the differences between the HOSS and Flat methods in the optimization of reduced MAPK model case. Based on Figure 7D, I would conclude that the cost values for the HOSS optimization are nearly the same as those for the Flat optimization, rather than being better.

In our view, Figure 7C and D demonstrate that HOSS optimization is effective and outperforms plain optimization. For the Mos-P species, the L2 cost is reduced by an order of magnitude compared to plain optimization. While the qualitative appearance of the fitted curves is similar, the reduced cost achieved by HOSS confirms the accuracy of our assertion.

Overall, I agree that the advantages of HOSS in execution time and the superiority of modular approaches over direct methods stem from the significant increase in optimization complexity as the problem size grows. However, it is difficult to conclude that the numerical experiments illustrate a gain in precision. Figure 2E and 2F indicate that HOSS by itself cannot fit the data well, and Figure 7D shows that HOSS has not improved precision too much when compared with Flat method. As an optimization tool, I believe it is crucial to clarify how accurately HOSS can fit the data and how much it can reduce the cost values compared to the Flat method.

We appreciate the reviewer's comments and suggestions, but we believe that our accuracy test presented in Figure 11 is comprehensive and statistically appropriate. Accuracy comparisons for complex optimization problems typically involve demonstrating that a method more effectively finds good solutions than other methods. In our case, due to potential conflicts within the datasets, the best solution (i.e., the one with the lowest cost) may not necessarily generalize the best. Therefore, it is appropriate to consider the distributions of both optimal and suboptimal solutions generated by each method, as we do in Figure 11. Figures 11 E, F, G, and H show that the cost distributions produced by HOSS are clearly distinct from those of flat methods, with HOSS consistently outperforming them. A strategy for testing optimization methods, similar to ours, was implemented in
Villaverde AF, Fröhlich F, Weindl D, Hasenauer J, Banga JR. Benchmarking optimization methods for parameter estimation in large kinetic models. Bioinformatics. 2019 Mar 1;35(5):830-838. doi: 10.1093/bioinformatics/bty736. PMID: 30816929; PMCID: PMC6394396.
Figure 1B in the cited paper illustrates the distribution of objective function values after a prescribed time, serving as the performance criterion.
